# A Robust Screen-Free Brain-Computer Interface for Robotic Object Selection

**DOI:** 10.3389/frobt.2020.00038

**Published:** 2020-03-31

**Authors:** Henrich Kolkhorst, Joseline Veit, Wolfram Burgard, Michael Tangermann

**Affiliations:** ^1^Brain State Decoding Lab, Department of Computer Science, University of Freiburg, Freiburg im Breisgau, Germany; ^2^Autonomous Intelligent Systems, Department of Computer Science, University of Freiburg, Freiburg im Breisgau, Germany; ^3^Cluster of Excellence BrainLinks-BrainTools, University of Freiburg, Freiburg im Breisgau, Germany; ^4^Toyota Research Institute, Los Altos, CA, United States

**Keywords:** brain-machine interface, screen-free brain-computer interface, subclass structure, human-robot interaction, event-related potentials, service robots

## Abstract

Brain signals represent a communication modality that can allow users of assistive robots to specify high-level goals, such as the object to fetch and deliver. In this paper, we consider a screen-free Brain-Computer Interface (BCI), where the robot highlights candidate objects in the environment using a laser pointer, and the user goal is decoded from the evoked responses in the electroencephalogram (EEG). Having the robot present stimuli in the environment allows for more direct commands than traditional BCIs that require the use of graphical user interfaces. Yet bypassing a screen entails less control over stimulus appearances. In realistic environments, this leads to heterogeneous brain responses for dissimilar objects—posing a challenge for reliable EEG classification. We model object instances as subclasses to train specialized classifiers in the Riemannian tangent space, each of which is regularized by incorporating data from other objects. In multiple experiments with a total of 19 healthy participants, we show that our approach not only increases classification performance but is also robust to both heterogeneous and homogeneous objects. While especially useful in the case of a screen-free BCI, our approach can naturally be applied to other experimental paradigms with potential subclass structure.

## 1. Introduction

Robotic service assistants can help impaired users gain autonomy by performing physical tasks, such as fetching objects or serving a meal. While the robots should perform low-level actions without supervision, the human should be able to exert high-level control, such as deciding which object to fetch and deliver. Non-invasive brain signals can be used to deliver such control commands—especially for users who cannot reliably communicate using other modalities. For example, event-related potentials (ERPs) form one class of possible control signals from electroencephalography (EEG). Traditionally, computer screens with graphical user interfaces present stimuli in order to elicit ERP responses and to map brain responses to application commands. However, the required association between elements of the user interface and objects or actions in the real world can be ambiguous (e.g., in the presence of multiple identical objects).

**Figure 1:**
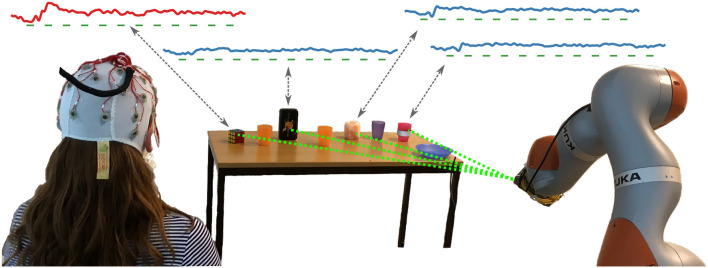
Setup of our screen-free BCI: While the user attends to her goal object (the Rubik's cube in this example), the robot sequentially highlights candidates in the environment using a laser pointer mounted adjacent to the end effector. As depicted by the grand averages in the top, the EEG responses to the highlighting stimuli (marked by green bars) differ between the target object of the user (the Rubik's cube, red curve) and the other non-targets (blue curves). However, the optical properties cause dissimilar responses across objects for both classes. Our approach is able to robustly handle these differences in brain responses when predicting the user goal from the EEG response to each highlighting.

Yet user goals in human–robot interaction are often related to tangible objects of the environment that the robot can manipulate. Hence, interaction with objects offers an alternative to screen-based selection. For instance, the robot can highlight candidate objects in the environment using a laser pointer as introduced in Kolkhorst et al. (2018). The ERP responses elicited by each laser highlighting allow to identify the target of a user's visual attention among multiple candidate objects (c.f., Figure 1). This novel approach avoids the indirection of an additional screen and permits changing environments while utilizing the robot that is needed anyhow for user assistance. Screen-free object selection was shown to work reliably in an online setting in Kolkhorst et al. (2018). Yet, the original evaluation was limited to candidate objects that shared similar surface properties and consequently looked similar upon illumination with the laser.

In more realistic application environments, possible candidate objects have *heterogeneous* optical properties. These could affect the elicited brain responses and lead to different ERP distributions across objects. In screen-based brain-computer interfaces (BCIs), such heterogeneous stimuli could be considered a—correctable—design flaw. In contrast, a high variance in surfaces and therefore stimuli is inherent to an in-the-scene highlighting approach and cannot be easily mitigated by modifying the experimental paradigm. Instead, it is desirable to address this problem from a machine-learning perspective.

One strategy is to treat each object—corresponding to a partition of the original classification task—as a separate subclass problem. A simple method would be to train separate classifiers for each subclass (e.g., object). However, as such a specialization entails reduced training data, it may be detrimental when applied to objects with similar optical properties. A more data-efficient treatment of subclass structure has been proposed by Höhne et al. (2016). While using traditional mean electrode potentials as ERP features, the authors proposed to regularize each subclass classifier toward other subclasses.

In this work, we address the problem of heterogeneous subclasses in the context of screen-free BCIs by training subclass-regularized classifiers in a Riemannian-geometry framework. Our contributions are threefold: First, we show that different object and stimulation properties lead to heterogeneous EEG responses in a screen-free BCI. Second, we propose to use subclass-regularized linear discriminant analysis (LDA) classifiers in a centered Riemannian tangent space to handle this heterogeneity. This can be viewed as a weighted incorporation of data from other subclasses. Third, we show on data from experiments with 19 participants observing homogeneous and heterogeneous stimuli that our approach improves classification performance compared to subclass-specific as well as subclass-agnostic classifiers while not deteriorating performance for non-relevant subclass information. Our improvements also hold for small amounts of training data.

This paper extends our earlier conference contribution (Kolkhorst et al., 2019b) in several ways: We corroborate the results of our earlier pilot study (which included 6 participants) on data from 19 participants. Additionally, we propose to center subclasses using parallel transport and regularize each classifier toward other objects rather than applying separate object-specific classifiers—allowing a more efficient use of data. We also provide a more comprehensive description of the approach and an extended discussion.

After reviewing related work in section 2, we describe our experimental paradigm—using a robot equipped with a laser pointer to elicit ERPs for candidate objects—in section 3.1. Subsequently, we explain our approach to handle subclass information in a Riemannian-geometry classification framework in section 3.2. After reporting results both regarding neurophysiology of responses and classification performance in section 4, we close with a discussion and conclusion in sections 5 and 6.

## 2. Related Work

As non-invasive brain-computer interfaces promise to offer communication capabilities to users who cannot reliably use other modalities, a wide range of command-and-control applications has been investigated. While multiple signal categories such as mental imagery can provide information, here we focus on experimental paradigms utilizing event-related potentials in response to a presented stimulus. Examples include spelling using visual (Sellers and Donchin, 2006; Hübner et al., 2017; Nagel and Spüler, 2019), auditory (Schreuder et al., 2010) or tactile (van der Waal et al., 2012) information and control of devices (Tangermann et al., 2009). In order to classify individual ERP responses, mean electrode potentials in suitable time intervals after the stimulus can be combined with linear classification methods (Blankertz et al., 2011). Recently, approaches based on deep learning (Schirrmeister et al., 2017; Behncke et al., 2018; Lawhern et al., 2018) or covariance representations leveraging Riemannian geometry (Barachant et al., 2013; Barachant and Congedo, 2014; Congedo et al., 2017) have gained popularity—both for ERPs and other signal classes—and can be considered state of the art.

Since the stimulus presentation is key for reliable analysis and classification of ERPs, consistent differences in presentation also affect the distribution of elicited ERPs. For example, variations in the target-to-target interval or habituation effects have been found to cause non-identically distributed ERP responses and affect classification performance (Citi et al., 2010; Hübner and Tangermann, 2017). These aspects can be viewed as *subclasses* of the data. Höhne et al. addressed the subclass structure in neuroimaging applications by training separate LDA classifiers for each subclass while regularizing them toward other subclasses using multi-target shrinkage (Bartz et al., 2014; Höhne et al., 2016). In principle, adapting subclass-specific classifiers to other subclasses can also be interpreted as a special case of transfer learning (e.g., Jayaram et al., 2016), which aims to improve performance by leveraging data from related tasks or datasets. In the context of Riemannian geometry (unsupervised), parallel transport of covariance matrices to a common point on the manifold (Zanini et al., 2018; Yair et al., 2019) as well as additional (supervised) geometric transformations (Rodrigues et al., 2019) have been proposed to reduce the differences in distributions between related datasets.

In the context of human-robot interaction—e.g., with service robots—the user should typically be able to deliver (high-level) commands to the robot (e.g., which object to fetch). Different modalities such as screens, speech (e.g., Shridhar and Hsu, 2018) or manually marking target objects using a laser pointer (Gualtieri et al., 2017) can be used to deliver these commands. However, impaired users might not be able to reliably control the robot using common modalities.

Brain signals as a feedback modality have also been used for command-and-control scenarios in robotics environments, e.g., to control wheelchairs or telepresence robots (Iturrate et al., 2009; Leeb et al., 2015), in fetch-and-carry tasks (Burget et al., 2017) or for grasp selection (Ying et al., 2018). These approaches typically use screens for stimulus presentation. While approaches based on mental imagery do not strictly require stimulus presentation, they would be limited in practice to only a small number of different commands in the absence of a mediating user interface.

A smaller number of publications utilized informative EEG signals from users who were passively observing a moving robot. Examples include the identification of erroneous actions (Salazar-Gomez et al., 2017; Behncke et al., 2018) or user preferences for robot motion (Iwane et al., 2019; Kolkhorst et al., 2019a). While this allows to infer user judgment of robotic actions, it requires the robot to first perform a candidate action (e.g., moving to the object with the highest prior probability). As goals of the user will often be outside of the robot's workspace, this strategy is typically more time-consuming than screen-based strategies. In addition, robotic motion is not instantaneous, which lowers the classification performance obtainable from event-locked evoked signals. Consequently, highlighting of objects with a laser pointer can be viewed as a combination of a natural user task with the ability to query many candidates and the precise stimulus timing known from traditional ERP paradigms.

## 3. Materials and Methods

In this section, we describe our screen-free BCI setup with heterogeneous objects as well as the data collection process. Subsequently, we present the covariance-based classification pipeline and specifically our approach to subclass-regularized classification of ERP signals in the Riemannian tangent space.

### 3.1. Screen-Free Stimulus Presentation Using Highlighting in the Environment

The idea of our screen-free BCI is to keep the user's attention on the environment while presenting stimuli—eliciting informative event-related potentials that allow decoding of the user goal. In our setup, the stimuli are presented by highlighting candidate objects with a laser pointer, which results in a temporally precise onset and an—ideally—salient stimulus.

#### 3.1.1. Experimental Setup for Robotic Object Selection

In order to highlight the candidate objects of the user, we used a KUKA iiwa robotic arm with a 1 mW green laser pointer mounted next to the end effector. As depicted in Figure 1, in our experiments the robot was positioned in front of a table with objects. Since robot movements are needed to orient the laser pointer to different objects, the robot highlighted the same object multiple times in a sequence lasting 3 s before switching to the next. For each highlighting, the laser pointer was turned on for 100 ms.

Highlighting poses for the robot were determined based on the positions of objects in the scene (in our ROS implementation, we expect a TF coordinate frame (Foote, 2013) for each object). In pilot experiments, we obtained these positions from a vision-based object detector and tracker (SimTrack, Pauwels and Kragic, 2015), which allows for the scene to change between different highlighting sequences. In the EEG experiments, we fixed object positions to reduce potential failures due to tracking errors. We obtained the desired robot configuration for highlighting an object based on the kinematics of the robot by minimizing required joint motion during the transition from the previous highlighting configuration while requiring the beam direction of the laser pointer to intersect with the object's position.

**Figure 2:**
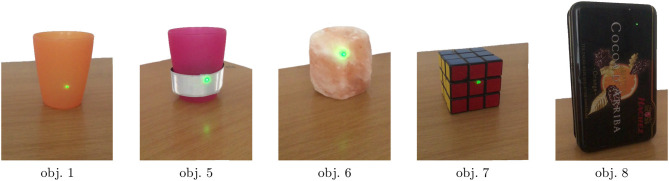
Close-up photos of candidate objects during highlighting in our experiments. The differing optical properties cause dissimilar appearances (using identical laser pointer illumination). Object 1 is a representative of the homogeneous objects (see Figure 1 for all objects), for which the highlighting appears similar. Objects 5 to 8 correspond to the heterogeneous objects. Note the reflection on the table surface for objects 5 and 6, and the low salience of the laser point at the top right of object 8.

As depicted in Figure 1, we used eight candidate objects as potential user goals: four *homogeneous* ones with similar surfaces (the same as in Kolkhorst et al., 2018), and four *heterogeneous* ones, for which we used everyday objects with differing optical surface properties. The different appearances of the highlightings with a laser pointer are depicted in Figure 2. The homogeneous objects (using obj. 1 as a representative) consisted of semi-transparent plastic, resulting in a salient spatially confined point. Despite varying shape, all homogeneous objects (three plastic cups and a plate; see Figure 1) resulted in similar stimulus intensities upon highlighting.

The heterogeneous objects caused different appearances of the laser upon illumination. Two objects (obj. 5 and obj. 6) induced large and salient points, whereas the laser points on obj. 7 and obj. 8 were less salient. Note that the materials and shapes of objects affected multiple optical properties. For example, obj. 5 (and, to a lesser degree, obj. 6) partially reflected the light, leading to an additional visible spot on the surface of the table. Although optical properties differed between objects, the effects (e.g., reflections) are not necessarily detrimental as they may increase stimulus salience. For an additional impression of the experiment setup and the appearance of stimuli, we refer the reader to the Supplementary Video.

We acquired the brain signals using a cap holding *N*_c_ = 31 Ag/AgCl gel-based passive EEG electrodes (EasyCap) positioned according to the extended 10–20 system (Chatrian et al., 1985, see Figure S2) with a nose reference. We kept channel impedances below 20 kΩ. The amplifier (Brain Products BrainAmp DC) sampled the EEG signals at 1 kHz.

#### 3.1.2. Experiments and Datasets

In this work, we report results from experiments with 19 healthy participants. The recorded EEG data is publicly available (Kolkhorst et al., 2020). Following the Declaration of Helsinki, we received approval by the Ethics Committee of the University Medical Center Freiburg and obtained written informed consent from participants prior to the session.

Each experiment consisted of multiple trials, in which the user had a constant goal object and four candidate objects (either the homogeneous or the heterogeneous ones) were highlighted. Each trial consisted of three repetitions, where one repetition corresponds to highlighting each candidate object in turn with a 3 s stimulus sequence. A schematic overview of the experiment structure can be found in Figure S1. We performed online experiments, i.e., after each trial we provided visual feedback on the decoded target object of the user. For the online feedback, we used a classifier without subclass handling which was trained at the beginning of the experiment (see Kolkhorst et al., 2018 for details on the online setup).

We asked the participants to put themselves in the condition of a user of an assistive robotic arm and that they could decide which object the robot should fetch by *attending* the object. We instructed participants to attend the goal object throughout the trial. While we did not mandate it, we expect that most participants maintained visual focus on the goal (i.e., used overt attention). We asked participants to sit relaxed and to minimize eye movements during a trial. In order to support the performance evaluation, we determined the goal object according to the experimental protocol and gave it as a cue to the participant prior to the start of every trial.

To investigate the influence of different object types and show applicability to varying stimulus-onset asynchronies (SOAs, the time between the start of subsequent laser highlightings), we performed three series of experiments: In the first, sessions with 7 participants were each split equally into two conditions: In each trial, either the four heterogeneous objects were highlighted using an SOA of 250 ms (the corresponding data is subsequently denoted by Het1) or the homogeneous objects were highlighted, also with an SOA of 250 ms (denoted by Hom1). In the second series with 6 participants—which has previously been used in Kolkhorst et al. (2019b)—each session was split into trials with heterogeneous objects and 250 ms SOA (Het2), and trials with homogeneous objects and an SOA of 500 ms (Hom2). The third series with 6 participants was originally described in Kolkhorst et al. (2018) and contains solely trials with the homogeneous objects and an SOA of 500 ms (Hom3). Hence, objects and stimulus parameters are identical between Het1 and Het2 as well as between Hom2 and Hom3. The SOAs affect the overlap between ERP responses to subsequent stimuli. Differences in SOA lead to variations in discriminability of individual stimuli, information transfer rate as well as usability (c.f., Höhne and Tangermann, 2012).

Each individual highlighting *k* can be associated with the target or non-target class *y*(*k*) ∈ {t, nt}. We investigated different definitions for subclasses of stimuli: The prime categorization is the mapping of stimuli to object instances *o*(*k*) ∈ {1, …, 8}. Since we performed multiple sequential highlightings for a single object before moving to the next, we can also identify each highlighting with the position within the stimulus sequence *q*(*k*) ∈ {1, …, 12}. As we expect most variation between the first and subsequent stimuli, stimulus positions can be aggregated to $\tilde{q}\left(k\right)$ with $\tilde{q}\left(k\right)\;=$ *initial* if *q*(*k*) = 1 and $\tilde{q}\left(k\right)=$ *subsequent* for *q*(*k*) > 1. For a more general notation, we use *j* to denote the index of a subclass (i.e., representing a value of either *o*, *q* or $\tilde{q}$ in this paradigm) and use *N*_sub_ for the number of subclasses (i.e., the number of unique values of *j* for a given subclass definition). The index set $K_{j}$ denotes the indices of highlightings belonging to this subclass. The number of highlightings for each of the data subsets and for each subclass in the different experiment conditions can be found in Table 1.

**Table 1:** Dataset Characteristics: Number of participants and stimuli for the different datasets, grouped by subclass definition.

**Subclass**	**n/a**			**Obj (**o**)**			**Stim (**$\tilde{q}$**)**		
**obj. type**	**het**	**hom**		**het**	**hom**		**het**	**hom**	
**SOA**	**250 ms**	**250 ms**	**500 ms**	**250 ms**	**250 ms**	**500 ms**	**250 ms**	**250 ms**	**500 ms**
No. of participants	13	7	12	13	7	12	13	7	12
Targets/subclass	864	864	648	216;216;216;216	216;216;216;216	162;162;162;162	72;792	72;792	108;648
Non-targets/subclass	2,592	2,592	1,938	648;648;648;648	648;648;648;648	484;484;484;484	216;2,376	216;2,376	323;1,938

*The group consisting of the first three columns (the subclass denoted by “n/a”) corresponds to the complete data, while the other two groups correspond to the data when using objects (“obj”, o) or stimulus position (“stim”, $\tilde{q}$) as a subclass definition. The columns for heterogeneous objects with an SOA of 250 ms apply to datasets Het1 and Het2, homogeneous objects with an SOA of 250 ms to Hom1 and the columns for homogeneous objects with an SOA of 500 ms to Hom2 and Hom3. The number of stimuli per subclass are separated by semicolons and correspond to a single experiment session. Note that the number of stimuli per subclass is uniform in the case of objects while it varies for the two subclasses based on $\tilde{q}$ (“stim”)*.

### 3.2. EEG Decoding

**Figure 3:**
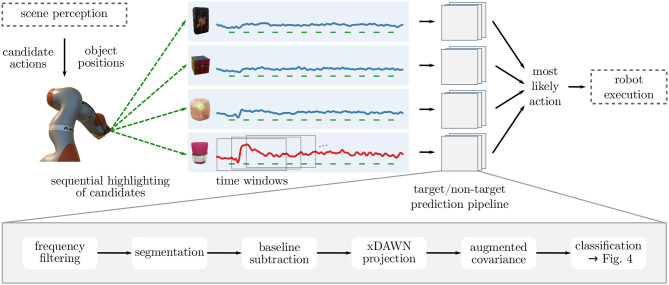
Overall architecture of the screen-free BCI: In order to identify user intentions, candidate objects—corresponding to possible actions of the robot—are highlighted sequentially. Time windows aligned to each stimulus are preprocessed and their covariance representation is used for classification (see lower part of the figure and Figure 4). Based on the predicted probabilities that the user attended the object in a given time window (i.e., whether it was the target), the most likely candidate is identified and the appropriate action is taken. In a future application setting, we see this pipeline combined with scene perception and object manipulation (marked by dashed boxes). In this work, however, we fixed candidate objects and gave purely visual feedback on the decoded target object rather than executing a grasp.

Identifying the goal object of the user reduces to a binary classification problem on the EEG data: Since we know the highlighted object for each brain response, we want to decode whether the user attended the highlighted object (for which we expect a *target* response) or not (*non-target* response). Hence, the target object can be predicted by choosing the object for which the target scores of the corresponding highlightings are highest. Figure 3 shows the overall processing pipeline for selecting the target from multiple candidates in the scene.

**Figure 4:**
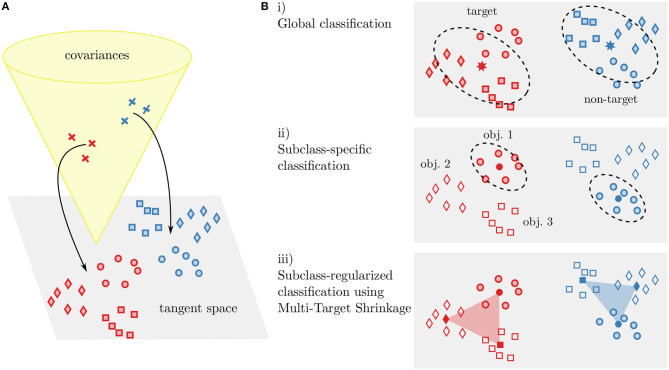
Illustration of our covariance-based classification approaches. Covariances $C_{k}\subset S_{++}$ are projected into the Riemannian tangent space ${T|}_{C^{m}}$ **(A)**. In the tangent space, classification is performed using linear discriminant analysis **(B)**. Either a single classifier is trained on the data (i, corresponding to *TS+LDA*), separate classifiers are trained for each subclass (ii, *sep. TS+LDA*), or the class means of subclass-specific classifiers are regularized toward other subclasses (iii, *cTS+reg-LDA*). This can be seen as a weighted inclusion of data from other subclasses. Note that for the latter case, we also perform centering by parallel transport of the members of each subclass in covariance space. In the figure, classes (target and non-targets) are indicated by color and subclasses (object instances) by different markers.

We use a classification pipeline based on Riemannian geometry: Covariances matrices of each time window are used as a signal representation, projected into the tangent space (TS) and classified using linear discriminant analysis (LDA). In order to handle heterogeneous subclasses in the data, we investigated three different classification approaches (as illustrated in Figure 4): The simplest way is to ignore subclass information and train classifiers on the pooled data (***TS+LDA***). Alternatively, specialized classifiers can be trained separately for each subclass (***sep. TS+LDA***). Third, we propose to perform subclass-specific centering and train specialized classifiers that are regularized toward other subclasses (***cTS+reg-LDA***), utilizing the full data.

#### 3.2.1. Segmentation and Preprocessing

As depicted in Figure 3, we perform preprocessing steps before estimating covariances: We filter the continuous EEG to the range of 0.50 Hz to 16 Hz before downsampling to 100 Hz. For classification, we extract one time window $X_{k}\in {\mathbb{R}}^{N_{\text{c}}\times N_{t}}$ of 1 s duration for each highlighting *k*, starting at the onset of the laser light (*N*_*t*_ = 101). To remove offsets, we subtract the mean activity of the preceding 200 ms. Note that most windows contain the response to multiple stimuli (four in the case of 250 ms SOA, two in the case of 500 ms). We reject windows in which the peak-to-peak amplitude exceeded 100 μV in any channel.

To capture the temporal course of the ERPs, we follow Barachant and Congedo (2014) and augment each window ***X***_*k*_ with prototype responses. This leads to a block structure of the covariance that also captures temporal information: Aside from the covariance of the measured signal, two blocks correspond to the cross-covariance of the signal with the ERP prototypes, providing information on the phase difference of signal and prototypes. As prototypes, we use the Euclidean mean ${\bar{X}}_{i}$ of responses for the target and non-target classes in the training data (*i* ∈ {t, nt}). To reduce dimensionality, we project the data based on spatial filtering components obtained from xDAWN decompositions (Rivet et al., 2009). We select two components per class corresponding to the largest eigenvalues, resulting in filter matrices $W_{i}\in {\mathbb{R}}^{2\times N_{\text{c}}}$. This leads to augmented windows

(1) $$\begin{array}{l} {\tilde{X}}_{k}=\left(\begin{array}{c} W_{\text{t}}{\bar{X}}_{\text{t}}\\ W_{\text{nt}}{\bar{X}}_{\text{nt}}\\ W_{\text{t}}X_{k}\\ W_{\text{nt}}X_{k} \end{array}\right)\in {\mathbb{R}}^{N_{c^{\prime }}\times N_{t}} \end{array}$$

with $N_{c^{\prime }}=8$ surrogate channels out of which the first four are the prototypes (these are constant across windows).

#### 3.2.2. Covariance-Based Decoding Pipeline

In this section, we give a brief description of our Riemannian geometry-based classification pipeline. For more details and motivation, we refer the reader to Pennec et al. (2006), Congedo et al. (2017), and Yger et al. (2017). For each augmented time window ${\tilde{X}}_{k}$ (i.e., each highlighting event), we calculate window-wise covariances $C_{i}\in S_{++}\left(N_{c^{\prime }}\right)$ that lie in the cone of $N_{c^{\prime }}\times N_{c^{\prime }}$ symmetric positive-definite matrices. Note that we regularize covariances using analytically determined shrinkage (Ledoit-Wolf). While Euclidean distances are ill-suited in $S_{++}$ (c.f., Yger et al., 2017)—hindering the use of standard classification approaches—each element ***C***^ref^ of the manifold can be associated with a tangent space ${T|}_{C^{\text{ref}}}$ at this point (Barachant et al., 2013).

Covariance matrices ***C*** are mapped to their tangent space representation ***S*** using $\text{Log}{\text{m}}_{C^{\text{ref}}}\left(C\right)=\text{logm}\left({\left(C^{\text{ref}}\right)}^{-1/2}C{\left(C^{\text{ref}}\right)}^{-1/2}\right)$. Here logm denotes the matrix logarithm. The matrix logarithm and the square root of a matrix $C\in S_{++}$ can be calculated by applying the corresponding scalar operation to the elements of the diagonal matrix ***Λ*** obtained from the decomposition ***C*** = ***Q*Λ*****Q***^*T*^. As a reference point, we use the Fréchet mean ***C***^*m*^ of the training data with regard to the affine-invariant Riemannian metric (c.f., Congedo et al., 2017). The reverse operation—mapping from ${T|}_{C^{\text{ref}}}$ to $S_{++}$—is denoted by $\text{Exp}{\text{m}}_{C^{\text{ref}}}$.

While not necessarily positive definite, tangent space matrices $S\in S\left(N_{c^{\prime }}\right)$ are symmetric and can hence be vectorized into $s\in {\mathbb{R}}^{N_{c^{\prime }}\left(N_{c^{\prime }}-1\right)/2}$ (c.f., Barachant et al., 2013). In principle, an arbitrary classifier can be used in the tangent space. In this work, we choose linear discriminant analysis (LDA). Note that in preliminary tests we found LDA to yield a classification performance similar to logistic regression [which was used in Kolkhorst et al. (2018)]. The weight vector of an LDA is given by $w_{\text{LDA}}=C_{\text{LDA}}^{-1}(\mu_{t}-\mu_{\text{nt}})$ for class means ***μ****_t_*, ***μ****_nt_* and the total within-class covariance matrix ***C***_LDA_. This classification pipeline—training a single classifier on the training data—is denoted by *TS+LDA*.

#### 3.2.3. Classifiers for Subclasses

In order to handle the heterogeneous subclasses in the data (primarily different objects), we explore the use of separate classifiers for each subclass. These can either be separate (using only data from a single subclass) or include regularization toward other subclasses (i.e., incorporating data from other subclasses).

As a first approach, we train separate subclass-specific classifiers (similar to Kolkhorst et al., 2019b). We apply the same classification approach as described above, but only on the data $\left\{X_{k}|k\in K_{j}\right\}$ of the corresponding subclass *j*. The Fréchet mean $C_{j}^{m}$ of the covariances belonging to *j* is used as the reference point for the tangent space projection of each subclass. Similarly, LDA classifiers are trained separately, leading to *N*_sub_ classifiers per participant. This classification pipeline is denoted by *sep. TS+LDA*.

##### 3.2.3.1. Subclass-regularized LDA

As a second way to leverage the subclass information, we adapt the regularization approach proposed by Höhne et al. (2016) (denoted by RSLDA in their paper): Rather than calculating the class means ***μ****_t_*, ***μ****_nt_* for the LDA classifier of subclass *j* only on the subset of the data corresponding to $K_{j}$, data from other subclasses *j*′≠*j* is also used by calculating a weighted class mean.

This can be formalized as multi-target shrinkage of the mean (MTS, Bartz et al., 2014; Höhne et al., 2016): For the classifier of subclass *j*, the shrunk mean of class *i* can be obtained by a convex combination of the corresponding class's means on all subclasses.

(2) $$\begin{array}{l} \mu_{i,j}^{MTS}\left(\alpha \right)=\left(1-\underset{j^{\prime }\neq j}{\sum }\alpha_{j^{\prime }}\right)\mu_{i,j}+\underset{j^{\prime }\neq j}{\sum }\alpha_{j^{\prime }}\mu_{i,j^{\prime }} \end{array}$$

Here, $\mu_{i,j}=\underset{\left\{k\in K_{j}|y\left(k\right)=i\right\}}{\sum }s_{k}$ corresponds to the mean of the vectorized tangent space representations of the given class and subclass. The coefficients ***α*** can be obtained by minimizing the expected mean square error, leading to a quadratic program based on the variance and bias of the different means. Intuitively, weights for other subclasses *j*′ should be small if distances between subclass means are large or if there is a high variance in the samples of *j*′ for the given class. For details, we refer the reader to Bartz et al. (2014) and Höhne et al. (2016).

##### 3.2.3.2. Parallel transport of subclasses

In order to regularize ***μ***_*i, j*_ toward the class mean of another subclass $\mu_{i,j^{\prime }}$, both should be located in the same tangent space. However, in the case of separate subclass classifiers, the reference points would also differ: The first class mean would be located in ${T|}_{{C^{m}}_{j}}$, while the second would be in ${T|}_{{C^{m}}_{j^{\prime }}}$. One possibility to address this would be to map the mean tangent vectors back into the manifold $S_{++}$ before projecting them in the correct tangent space:

(3) $$\begin{array}{l} \mu_{i,j}^{MTS}\left(\alpha \right)=\left(1-\underset{j^{\prime }\neq j}{\sum }\alpha_{j^{\prime }}\right)\mu_{i,j}+\underset{j^{\prime }\neq j}{\sum }\alpha_{j^{\prime }}\text{Log}{\text{m}}_{{C^{m}}_{j}}\text{Exp}{\text{m}}_{{C^{m}}_{j^{\prime }}}\mu_{i,j^{\prime }} \end{array}$$

As an alternative, we center each subclass using parallel transport on $S_{++}$ before tangent space projection, which has previously been proposed in the context of transfer learning (c.f., Zanini et al., 2018; Yair et al., 2019): A symmetric positive definite matrix (e.g., of subclass *j*) can be transported along the geodesic from ${C^{m}}_{j}$ to the identity using $\Gamma_{{C^{m}}_{j}\to I}\left(C\right)={\left({C^{m}}_{j}\right)}^{-1/2}C{\left({C^{m}}_{j}\right)}^{-1/2}$. Afterwards, ${C^{m}}_{j}=I$ for all subclasses *j* and the matrix logarithm and matrix exponential in Equation 3 cancel since all means are located in the same tangent space. Hence, Equation 3 reduces again to Equation 2. We denote the resulting classification pipeline—consisting of centering subclasses using parallel transport combined with subclass-regularized LDA classifiers—with *cTS+reg-LDA*. An overview of all classification approaches can be found in Figure 4.

#### 3.2.4. Evaluation

For each participant, classifiers were trained and tested in a five-fold chronological cross-validation. In order to evaluate the influence of data set size, we also report results on 33 % of the data (we used the first of three repetitions in each trial). Note that due to the interleaved design, data for both different objects and dataset sizes is temporally balanced within each experiment session. We evaluated the classification performance using the area under the receiver operating characteristic (AUC). The decoding pipeline was implemented in Python, building upon MNE-Python (Gramfort et al., 2013), scikit-learn (Pedregosa et al., 2011) and pyRiemann (Barachant and Congedo, 2014).

## 4. Results

In this section, we report participants' feedback on the paradigm before describing the influence of subclasses—different objects and position in stimulation sequence—on the elicited ERPs. Subsequently, we report classification performances for the different proposed subclass handling strategies.

### 4.1. Behavioral Feedback From Participants

In order to get insight into the feasibility and usability of the screen-free approach from a user perspective, we gathered feedback from participants in post-session questionnaires. Participants reported that the task induced a low stress level (24±19 on a visual analog scale from “relaxed,” which is represented by 0, to “stressed,” represented by 100) and required low mental demand (24±16 on a scale from “easy” to “demanding”). While answers reveal medium required effort (46±25 on a scale from “few” to “much”), verbal feedback from participants indicated that this was influenced by our instruction to avoid blinking. Overall, participants were satisfied with their task performance (76±19 on a scale from “unsatisfied” to “satisfied”). Answers of individual participants can be found in Figure S3.

### 4.2. Grand Average Responses to Laser Highlighting

The highlighting of objects with a laser pointer elicited various event-related potentials (ERPs) starting approximately 100 ms after stimulus onset. Similar to common screen-based visual ERP paradigms, responses appear to be a combination of early sensory and later cognitive components (c.f., Blankertz et al., 2011). Class-discriminative differences between target and non-target responses could be observed from approximately 200 ms after the onset of highlighting.

**Figure 5:**
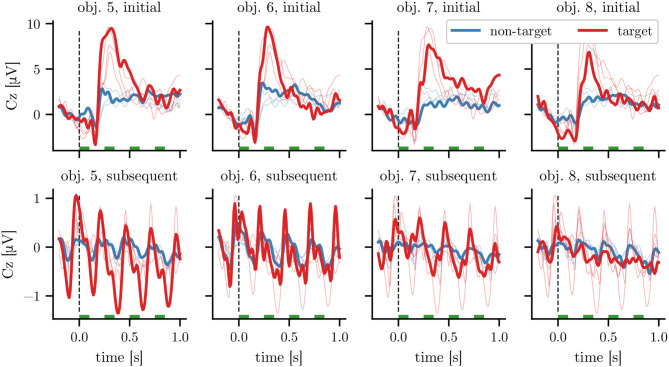
Grand average responses at electrode Cz for heterogeneous objects. Each column corresponds to the responses to the highlighting of a single object. The top row depicts the response to the initial stimulus of a 3 s stimulation sequence, while the bottom row depicts the average response to the 11 subsequent ones (see Figure 1 for averages over the full stimulation sequence). Time 0 corresponds to the onset of highlighting, with all stimulation intervals marked with green bars. For ease of comparison, the averages for other objects of the same row are shown with thin lines. The averages are calculated on the combined data of the 13 participants of datasets Het1 and Het2 using an SOA of 250 ms. The target (non-target) averages are calculated based on 234 (702) and 2,574 (7,722) time windows for the plots in the top and bottom row, respectively (before artifact rejection).

Looking separately at the grand average ERPs for different objects and stimulus positions within the sequence on the combined data of Het1 and Het2 as depicted in Figure 5, we can observe *heterogeneous responses* to these different subclasses: First, the initial highlighting of each sequence (depicted in the top row) resulted in an ERP with a higher amplitude—specifically around 300 ms, which is in line with the expected P300 response for target stimuli—than subsequent highlightings (bottom row; note the different axis limits). Second, we find that the ERPs differed between the four heterogeneous objects, both in amplitude and waveform. For example, amplitudes of both target and non-target ERPs for obj. 7 and obj. 8 were smaller than for the other objects. We observed, that latencies for obj. 8 varied stronger between participants than for other objects (data not shown). In contrast, differences in waveform between objects were smaller for the homogeneous objects in Hom1, Hom2, and Hom3, while the amplitude differences between the responses corresponding to first and subsequent stimuli are consistent with the differences for heterogeneous ones. The corresponding grand average plots for dataset Hom1 can be found in Figure S4.

### 4.3. Classification Results

Next, we investigate the classification performance on individual time windows corresponding to a single highlighting event. After comparing the subclass-agnostic classification performance (*TS+LDA*) on different object types, we report results on using separate subclass-specific classifiers (*sep. TS+LDA*) and the proposed subclass-regularized classifiers (*cTS+reg-LDA*).

First, we investigate how the difference between homogeneous and heterogeneous objects translates into classification performance. For this, the data of the 7 participants in datasets Het1 and Hom1 is well-suited, since they observed both heterogeneous and homogeneous objects with an SOA of 250 ms. We find that classification using the *TS+LDA* pipeline (i.e., disregarding subclass information) worked well for all participants and would be adequate for control. Yet on the time windows corresponding to heterogeneous objects, this pipeline achieved an AUC of 0.82 compared to 0.91 for homogeneous objects. This shows that the heterogeneity of objects described in the previous subsection also translates into reduced classification performance (see Table 2 for results on the different data subsets).

**Table 2:** Performance of the different classification approaches on the evaluated datasets.

	**Obj. type**	**het**		**hom**			
	**SOA**	**250 ms**		**250 ms**		**500 ms**	
	**data size**	**33%**	**100%**	**33%**	**100%**	**33%**	**100%**
**Subclass**	**classifier**						
n/a	TS+LDA	0.79 ± 0.09	0.82 ± 0.07	0.88 ± 0.07	0.91 ± 0.06	0.79 ± 0.10	0.84 ± 0.07
Obj (*o*)	sep. TS+LDA	0.76 ± 0.08	0.83 ± 0.06	0.80 ± 0.11	0.89 ± 0.07	0.71 ± 0.09	0.80 ± 0.08
	cTS+reg-LDA	**0.82** ±**0.08**	**0.86** ±**0.05**	**0.89** ±**0.06**	**0.92** ±**0.05**	0.79 ± 0.10	**0.85** ±**0.07**
Stim ($\tilde{q}$)	sep. TS+LDA	0.78 ± 0.08	0.82 ± 0.07	0.87 ± 0.07	0.91 ± 0.05	0.78 ± 0.09	0.84 ± 0.06
	cTS+reg-LDA	0.79 ± 0.09	0.82 ± 0.07	0.88 ± 0.07	0.92 ± 0.06	**0.80** ±**0.10**	0.85 ± 0.07

*Rows correspond to different classifiers, trained on the given subclass definition (“n/a” corresponds to training a single subclass-agnostic classifier). Columns correspond to the different datasets (see Table 1 for the sample counts using 100 % of the data). Performance is reported as the mean and standard deviation of the area under the receiver operating characteristic (AUC, higher is better). For each dataset, the bold AUC values highlight the classifier with the best performance*.

**Figure 6:**
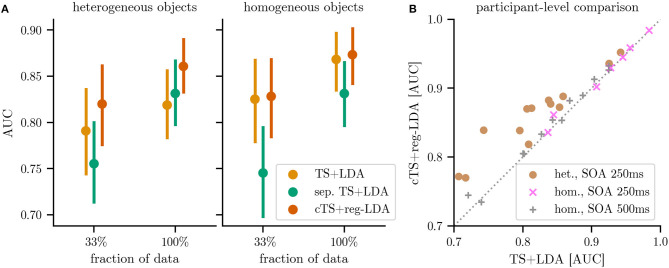
Performance of the different classification pipelines for classifying individual responses: Riemannian tangent space classification ignoring subclass information (*TS+LDA*), separate tangent space classifiers for each subclass (*sep. TS+LDA*) and the proposed separate subclass-regularized classifiers in centered tangent spaces (*cTS+reg-LDA*). For the latter two approaches, each object corresponds to a separate subclass. **(A)** shows the results for heterogeneous objects (combining the 13 participants of datasets Het1 and Het2) and for homogeneous objects (combining the 19 participants of datasets Hom1, Hom2, and Hom3). **(B)** plot compares the performance of *cTS+reg-LDA* and *TS+LDA* on the level of individual participants (colors indicate differing object types and SOA). Markers above the diagonal indicate that the proposed *cTS+reg-LDA* outperforms *TS+LDA*. Results are reported using the area under the receiver operating characteristic (AUC, higher is better) and error bars correspond to bootstrapped 95 % confidence intervals. Detailed results can be found in Table 2.

Using separate classifiers (*sep. TS+LDA*) for every object *o* to handle the heterogeneity, we observe improvements when ample training data is available: As depicted in Figure 6A, AUC performance on Het1 and Het2 (13 participants) improved slightly to 0.83 compared to 0.82 for the object-agnostic classifier when training on 100 % of the data. However, these improvements vanished if not enough (subclass-specific) training data was available: Using only 33 % of the data, performance dropped to 0.76 for separate subclass-specific classifiers compared to 0.79 for the subclass-agnostic classification. This reflects the smaller amount of training data (c.f., Table 1).

Using the proposed subclass-regularized classifiers in a centered tangent space (*cTS+reg-LDA*), we can observe improved performance over the baseline classifiers in the presence of heterogeneous objects: As the regularization takes into account information of other subclasses (i.e., objects *o*), *cTS+reg-LDA* resulted in gains over *TS+LDA* on the reduced data (mean AUC of 0.82 vs. 0.79) and the full data (0.86 vs. 0.82, a stronger improvement than *sep. TS+LDA*). Equally important, the proposed approach is also applicable to data where we do not expect a strong subclass structure: As depicted in Figure 6, when training subclass-regularized object classifiers on the full data of homogeneous objects, the proposed approach achieved a mean AUC of 0.87 and slightly outperformed *TS+LDA*, whereas training separate classifiers resulted in a drop to 0.83.

Using the stimulus positions $\tilde{q}$ as subclasses resulted in smaller effects: For *sep. TS+LDA*, we observed performance similar to a global classifier on the full data and a small deterioration on 33 % of the data. On the other hand, *cTS+reg-LDA* performed better or on the same level as *TS+LDA* when using the stimulus position ($\tilde{q}$) as subclasses. Detailed results for the separate subclasses and datasets can be found in Table 2.

Examining individual participants (as depicted in panel B of Figure 6), classification results using *cTS+reg-LDA* with objects as subclasses were better than the ones using *TS+LDA* for all 13 participants in the case of heterogeneous objects (Het1 and Het2) as well as for 14 of 19 participants in the case of homogeneous objects (Hom1, Hom2, and Hom3). For heterogeneous objects, the proposed approach resulted in a median absolute improvement in AUC of 0.04 across participants (minimal and maximal improvements of 0.01 and 0.10, respectively). To test the significance of differences in AUC between *cTS+reg-LDA* and *TS+LDA* in the presence of different object types and SOAs, we used a two-sided Wilcoxon signed-rank test at significance level α = 0.05 with a conservative Holm-Bonferroni correction. We can reject the null hypothesis both for heterogeneous objects with an SOA of 250 ms (adjusted *p* = 0.004) and—interestingly—also for homogeneous objects with an SOA of 500 ms (*p* = 0.030). We could not reject it for the 7 participants in the setting with homogeneous objects and an SOA of 250 ms (*p* = 0.398). As expected, effect sizes are larger in the presence of heterogeneous objects than for homogeneous ones (c.f., Figure 6).

**Figure 7:**
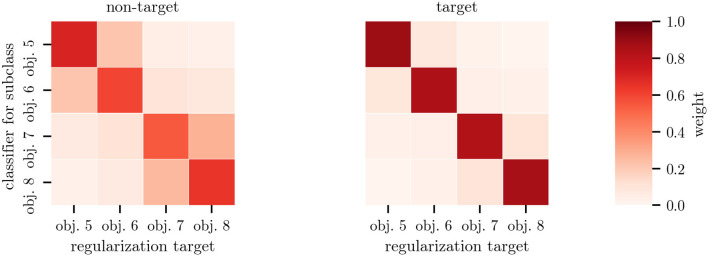
Mean regularization weights ***α*** for heterogeneous objects. Each row corresponds to the weights of a single subclass-specific classifier. On the left, regularization weights for the mean of the non-target class are shown whereas the corresponding weights for the target class are shown on the right. Entries can be viewed as sample weights of the data of the corresponding subclass. Note the object similarities indicated by the block structure in this figure compared to the stimulus appearances in Figure 2. The weights have been averaged across all participants of datasets Het1and Het2 with classifiers trained on 100 % of the data.

The weights ***α*** of the subclass regularization allow introspection into the classification: As depicted in Figure 7, the weights for heterogeneous objects show a clear block structure, with obj. 5 and obj. 6 as well as obj. 7 and obj. 8 being regularized to each other—reflecting similarities in the optical properties (c.f., Figure 2) and average responses (c.f., Figure 5). The classifier means for the non-target class were regularized more than the target ones. Regularization toward other subclasses was stronger when only 33 % of data was available. When applying *cTS+reg-LDA* to homogeneous objects, we also observed larger weights for other subclasses, indicating a smaller influence of the subclass structure on the classifier means (c.f., Figure S5).

## 5. Discussion

Building a screen-free BCI for robotic object selection by highlighting objects in the environment removes a level of indirection for the user. However, this benefit in terms of usability comes at the price of reduced stimulus homogeneity: When optical properties across candidate objects vary—as frequently encountered in real-world environments—the differences in appearance of the laser highlighting result in different feature distributions. While this can partly be attributed to a varying salience of stimuli, it is hard to mitigate by modifying the experimental design since we do not want to constrain or exchange the objects. In principle the laser parameters could be automatically adapted to different surfaces, yet this would pose a substantial challenge in practice. Whereas vision-based approaches can model optical properties such as the diffuse reflectance of surfaces (e.g., Krawez et al., 2018), we encountered combinations of translucency as well as diffuse and specular reflections.

Hence, we decided to approach this problem from a machine-learning perspective by modeling object instances as subclasses and training separate subclass-regularized classifiers that combine data from different subclasses in a weighted manner. We achieved strong performance gains by combining multi-target shrinkage of the mean (as proposed in Höhne et al., 2016) with both subclass-specific centering using parallel transport and the state-of-the-art performance of covariance-based Riemannian tangent space classifiers. Our approach is applicable to arbitrary subclasses and experimental paradigms. Note that the information about subclasses (e.g., objects) is readily available at test time. We found that the proposed pipeline significantly outperformed the baseline in the case of relevant subclasses, while being robust to irrelevant subclasses. Notably, we could observe smaller performance gains even when distributions do not substantially differ between subclasses (e.g., homogeneous objects). Performance did not deteriorate in the presence of a large amount of subclasses (e.g., for every position *q* in the stimulus sequence, data not shown). The effect sizes—gains of three or more points in AUC compared to ignoring the subclass information—were substantial and are relevant in practical applications. Introspection of the learned regularization weights (Figure 7) as a measure of subclass similarity mirrors the differences in visual appearance of the highlighting (Figure 2). While ERP amplitudes differed based on the position of a stimulus in the sequence (initial vs. subsequent), using this as a subclass resulted in smaller improvements in classification performance. This indicates that the Riemannian classification pipeline may be more robust to changes in amplitude rather than ERP waveform.

Exploring possible alternative classification approaches, in additional experiments (data not shown), we found the proposed specialized (i.e., subclass-regularized) classifiers to achieve higher classification performance than using subclass-specific normalization with a global classifier: While we found that subclass-specific centering of covariance matrices using parallel transport already improved classification, performance was lower than using our proposed approach (especially with sufficient training data). While additional geometric transformations [such as rotation to another subset of the data as proposed by Rodrigues et al. (2019)] could in principle be an alternative to the convex combination of subclass means, we observed reduced performance on our data. Applying subclass-regularized LDA on features based on mean electrode potentials in suitable time intervals (as reported in Höhne et al., 2016; results not shown) performed consistently worse than using Riemannian tangent space features (which matches the results in Kolkhorst et al., 2018).

A limitation of our current approach is the assumption that we have observed all subclasses in the training data. While this would likely not hold in practice, the subclass of a novel object could be assigned either based on visual similarity to known objects or using proximity of EEG signals in covariance space. Generally, it could be useful to use clusters of objects with similar optical properties as subclasses in the presence of a large number of objects. While we performed the analyses in this paper in an offline manner, the approach is applicable online. Compared to the subclass-agnostic classifiers during the online experiments (c.f., Kolkhorst et al., 2018), the additional computational burden of centering matrices is small, hence we are confident that results would translate to an online application.

The use of screen-free stimuli is not limited to specific stimulation parameters. In this work, we opted for stimulation aimed at eliciting ERPs as different candidate objects were highlighted sequentially rather than in parallel. The two representative SOAs in our experiments indicated robustness to different stimulus parameters. It would also be interesting to evaluate parallel screen-free stimuli with a higher frequency—more closely resembling broadband (Thielen et al., 2015) or steady-state (e.g., Chen et al., 2015) visual evoked potentials—as it is likely that different optical properties would also induce heterogeneous responses in such a setting. In this work, we used a constant stimulation sequence length for simplicity, yet information transfer rate could be increased by committing to a goal once a required confidence has been reached (i.e., dynamic stopping), which would also increase robustness to non-control states (Schreuder et al., 2013; Nagel and Spüler, 2019).

The proposed *cTS+reg-LDA* classification approach is also applicable outside of our screen-free BCI setting. While here the problem of non-identically distributed ERP responses induced by subclasses is especially relevant, subclasses are also frequently encountered in traditional, screen-based visual or auditory stimulus presentation. For example, varying locations of stimuli or different target-to-target intervals can also lead to dissimilar subclasses (c.f., Höhne et al., 2016; Hübner and Tangermann, 2017). Furthermore, the small improvements of our approach on homogeneous objects indicate that subclass information can be helpful even when no differences between subclasses are expected. Based on the found robustness it can be applied without risking a decline in classification performance.

Considering screen-free stimulus presentation in general, we view it as a building block that can be integrated into assistive human–robot interaction scenarios. Given that a robot is available to assist the user, it can also be used to present stimuli corresponding to possible assistive actions. As examples, it can be adapted to arbitrary goals that are related to spatial locations (e.g., where to place objects or how to avoid an obstacle) or it could be used for interactive teaching of the robot (e.g., where to grasp an object). Illuminating objects with a robot makes screen-free stimuli feasible in a changing environment with novel objects, as opposed to using active light sources on candidates. Combining the screen-free BCI with computer vision and manipulation modules (c.f., Figure 3), we envision that candidate (manipulation) actions are determined based on detected object affordances or anticipated user commands in a scene (e.g., Kaiser et al., 2016; Koppula and Saxena, 2016). As actions can be translated to appropriate highlightings of the corresponding objects, our BCI paradigm can then be used to choose between the candidates. Consequently, a screen-free BCI can be seen as a disambiguation module giving users direct control in a shared-autonomy setting.

## 6. Conclusion

In this work, we presented a screen-free brain-computer interface for an object selection task that allows robust decoding of user goals. Using the robot to present stimuli by highlighting objects in the environment with a laser pointer avoids the indirection of a graphical user interface but results in heterogeneous responses to objects in realistic environments. Our approach addresses this by training specialized classifiers for the object subclasses that are regularized based on the data of other objects.

In extensive experiments with 19 participants, we show that different optical properties of candidate objects induced distinct distributions of the corresponding brain responses. We find that our approach significantly improved classification performance in the presence of heterogeneous objects while not deteriorating in the presence of homogeneous ones. This increased robustness enables the application of screen-free BCIs in more diverse environments.

For future work, it would be interesting to also incorporate vision-based information on stimulus similarity for novel objects as well as increase communication bandwidth and applicability by using dynamic stimulus sequences and hierarchical goal selection.

## Data Availability Statement

The dataset recorded for this study (Freiburg Screen-free BCI for Robotic Object Selection: EEG Dataset) is publicly available at http://dx.doi.org/10.17605/OSF.IO/JZS7D.

## Ethics Statement

The studies involving human participants were reviewed and approved by the Ethics Committee of the University Medical Center Freiburg. The participants provided their written informed consent prior to participating in this study.

## Author Contributions

HK and MT contributed conception and design of the study. WB and MT acquired funding. HK and JV performed the EEG experiments, implemented software, and performed the analyses. HK wrote the original draft. JV, WB, and MT reviewed and edited the draft.

### Conflict of Interest

WB is affiliated with the Toyota Research Institute, Los Altos, USA. The remaining authors declare that the research was conducted in the absence of any commercial or financial relationships that could be construed as a potential conflict of interest.
