# Supplementary material for: A Robust Screen-Free Brain-Computer Interface for Robotic Object Selection
Source: Front Robot AI. 2020 Mar 31;7:38. doi: 10.3389/frobt.2020.00038 (PMC7806045; doi:10.3389/frobt.2020.00038)
Supplement: Supplementary file 1 [file Data_Sheet_1.pdf]

Supplementary Material for
“A Robust Screen-Free Brain-Computer Interface for Robotic Object Selection”

Henrich Kolkhorst *, Joseline Veit *, Wolfram Burgard and Michael Tangermann

This document contains supplementary figures for Kolkhorst et al. (2020).

1 SUPPLEMENTARY FIGURES

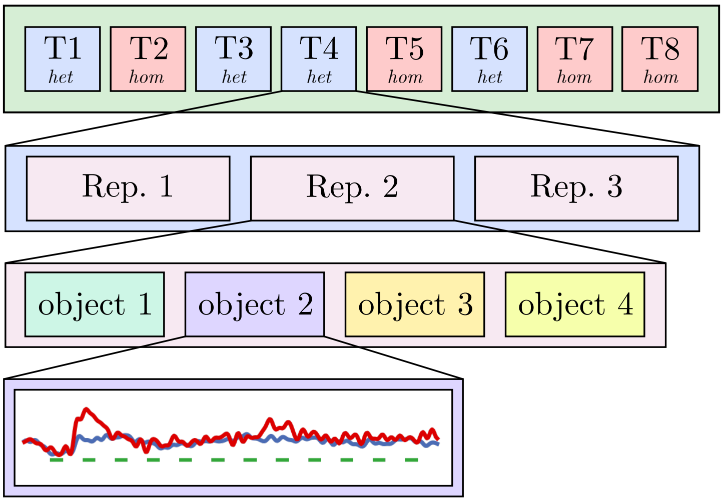

Figure S1. Structure of the experiments. Each experiment consisted of multiple blocks with each block being composed of multiple trials (denoted by T). Trials were assigned pseudo-randomly to either homogeneous or heterogeneous candidate objects while keeping trials of both types balanced in each block. The target object was constant within one trial. Each trial consisted of three repetitions. In each repetition, every candidate object was highlighted using a stimulation sequence of $3\,\mathrm{s}$ (i.e., 12 stimuli in the case of a stimulus-onset asynchrony of $250\,\mathrm{ms}$). The figure has been adapted from Kolkhorst et al. (2019).

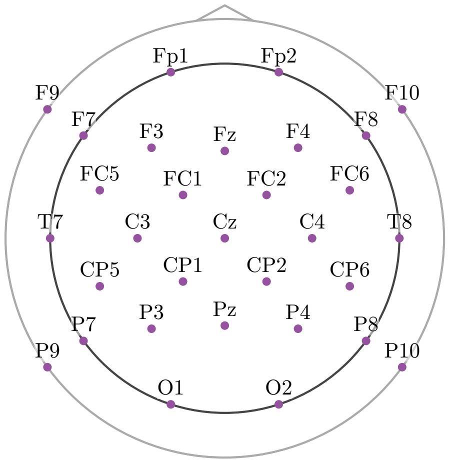

Figure S2. Electrode layout used in the experiments. This figure shows a top view of a participant's head. The triangle indicates the participant's nose. 31 Ag/AgCl gel-based passive EEG electrodes were placed according to the extended 10–20 system (Chatrian et al. (1985)). Signals were referenced to an electrode on the nose.

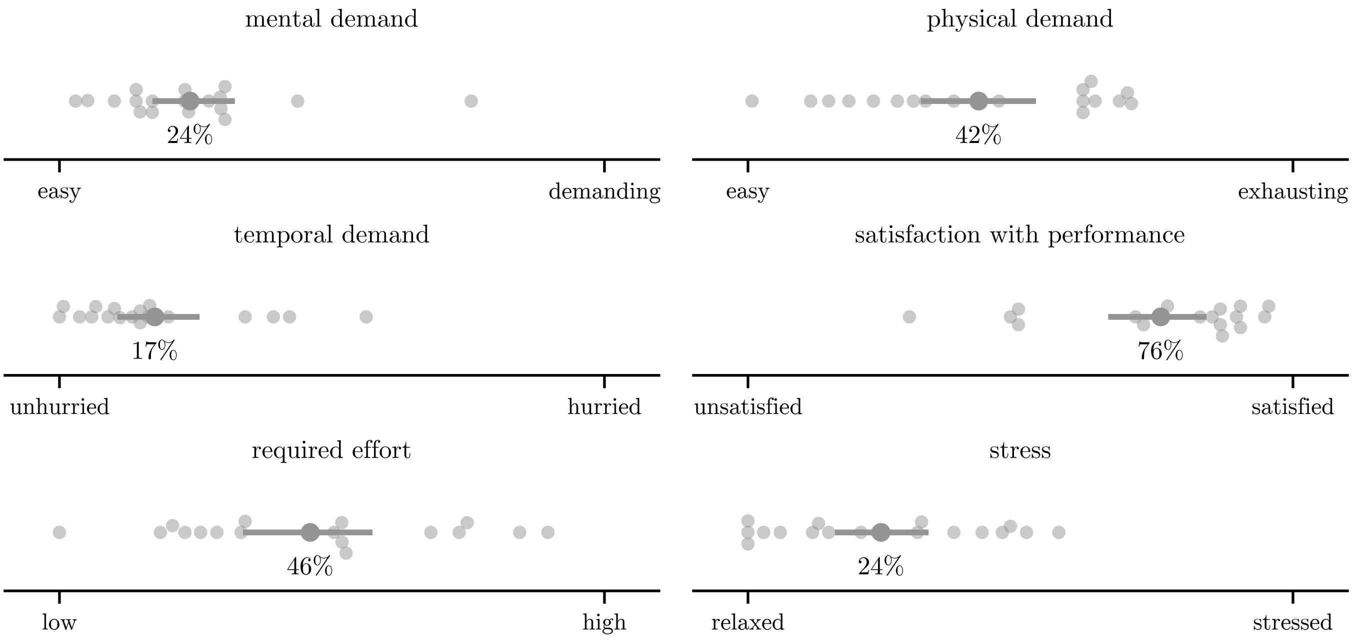

Figure S3. Participants' judgment of task and workload based on post-session questionnaire responses. Responses were recorded using visual analog scales. Note that responses were recorded with German questions and scale labels and have been translated to English for this figure. The opaque markers and bars denote mean responses with bootstrapped 95 % confidence intervals, while the semi-transparent markers correspond to answers by individual participants.

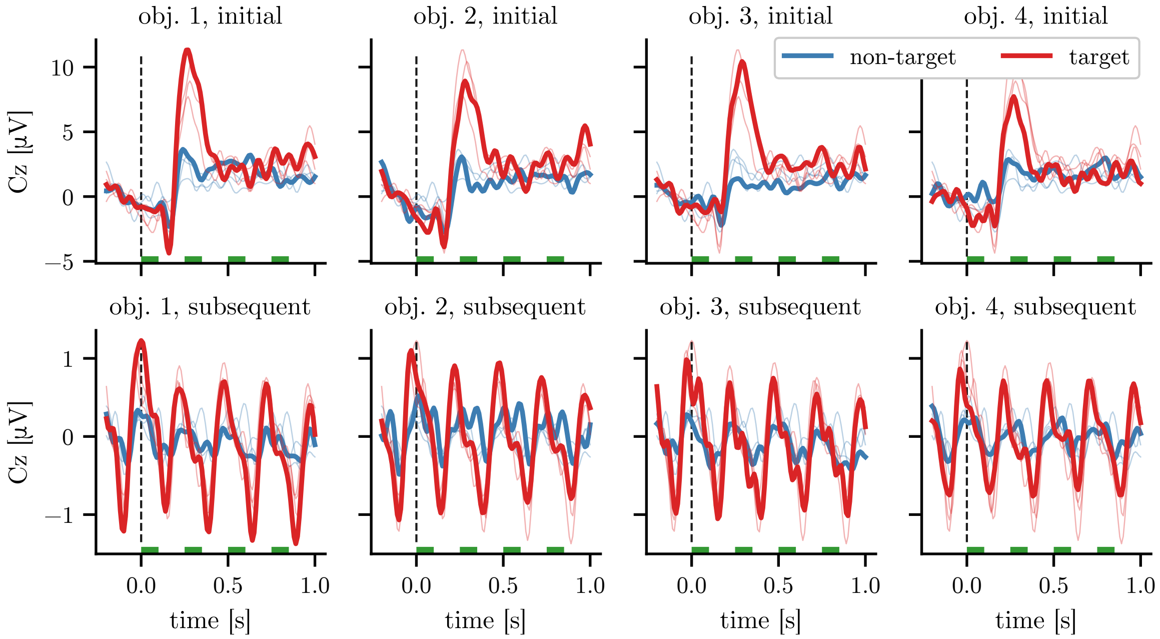

Figure S4. Grand average response at electrode Cz for homogeneous objects. Each column corresponds to the responses to the highlighting of a single object. The top row depicts the response to the initial stimulus of a 3 s stimulation sequence, while the bottom row depicts the average response to the 11 subsequent ones. Time 0 corresponds to the onset of highlighting, with all stimulation intervals marked with green bars. For ease of comparison, the averages for other objects of the same row are shown with thin lines. The averages are calculated on the data of the 7 participants of dataset HOM1 using a stimulus-onset asynchrony of 250 ms.

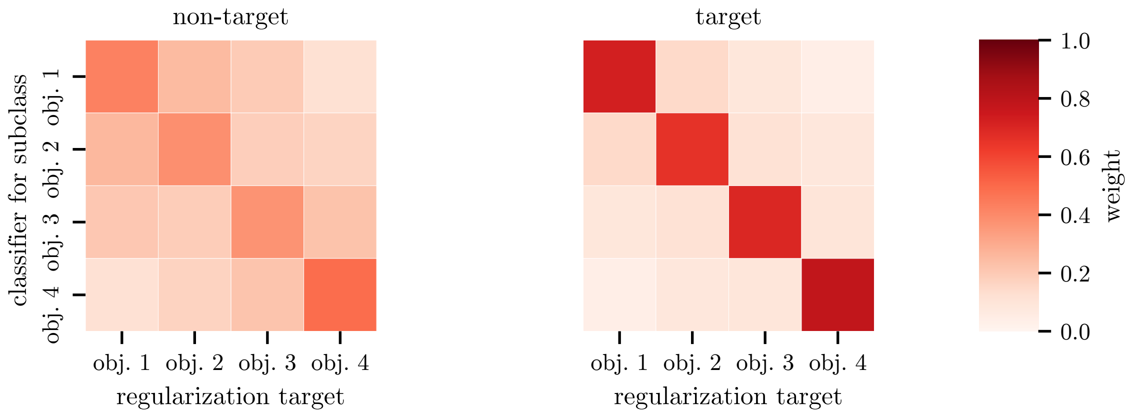

Figure S5. Mean regularization weights α for homogeneous objects. Each row corresponds to the weights of a single subclass-specific classifier. On the left, regularization weights for the mean of the non-target class are shown whereas the corresponding weights for the target class are shown on the right. Entries can be viewed as sample weights of the data of the corresponding subclass. The weights have been averaged across all participants of datasets HOM1, HOM2 and HOM3 with classifiers trained on 100 % of the data.

REFERENCES

Chatrian, G. E., Lettich, E., and Nelson, P. L. (1985). Ten Percent Electrode System for Topographic Studies of Spontaneous and Evoked EEG Activities. *American Journal of EEG Technology* 25, 83–92. doi:10.1080/00029238.1985.11080163

Kolkhorst, H., Veit, J., Burgard, W., and Tangermann, M. (2019). Heterogeneity of Event-Related Potentials in a Screen-Free Brain-Computer Interface. In *Proceedings of the 8th Graz Brain-Computer Interface Conference 2019* (Graz, Austria), 228–233. doi:10.3217/978-3-85125-682-6-42

Kolkhorst, H., Veit, J., Burgard, W., and Tangermann, M. (2020). A Robust Screen-Free Brain-Computer Interface for Robotic Object Selection. *Front. Robot. AI* 7. doi:10.3389/frobt.2020.00038. Research Topic: Advances in the Integration of Brain-Machine Interfaces and Robotic Devices
